# Comprehensive Review Regarding Mercury Poisoning and Its Complex Involvement in Alzheimer’s Disease

**DOI:** 10.3390/ijms23041992

**Published:** 2022-02-11

**Authors:** Emanuela Paduraru, Diana Iacob, Viorica Rarinca, Angelica Rusu, Roxana Jijie, Ovidiu-Dumitru Ilie, Alin Ciobica, Mircea Nicoara, Bogdan Doroftei

**Affiliations:** 1 Doctoral School of Geosciences, Faculty of Geography and Geology, Alexandru Ioan Cuza University of Iasi, No 20A, Carol I Avenue, 700505 Iasi, Romania; emanuela.paduraru@student.uaic.ro (E.P.); iacob.diana@student.uaic.ro (D.I.); rarinca_viorica@yahoo.com (V.R.); rusu_angelica@yahoo.com (A.R.); 2 Department of Exact and Natural Sciences, Institute of Interdisciplinary Research, Alexandru Ioan Cuza University of Iasi, No 20A, Carol I Avenue, 700505 Iasi, Romania; roxanajijie@yahoo.com; 3 Department of Biology, Faculty of Biology, Alexandru Ioan Cuza University of Iasi, No 20A, Carol I Avenue, 700505 Iasi, Romania; alin.ciobica@uaic.ro; 4 Center of Biomedical Research, Romanian Academy, No 8, Carol I Avenue, 700506 Iasi, Romania; 5 Academy of Romanian Scientists, No 54, Independence Street, Sector 5, 050094 Bucharest, Romania; 6 Faculty of Medicine, University of Medicine and Pharmacy Grigore T. Popa, No 16, University Street, 700115 Iasi, Romania; bogdandoroftei@gmail.com

**Keywords:** mercury poisoning, neurological toxicity, Alzheimer’s disease, oxidative stress, apoptosis, autophagy, gut microbiota, fertility

## Abstract

Mercury (Hg) is considered one of the most widespread toxic environmental pollutants, which seems to have multiple effects on organisms even at low concentrations. It has a critical role in many health problems with harmful consequences, with Hg primarily targeting the brain and its components, such as the central nervous system (CNS). Hg exposure was associated with numerous CNS disorders that frequently trigger Alzheimer’s disease (AD). Patients with AD have higher concentrations of Hg in blood and brain tissue. This paper aims to emphasize a correlation between Hg and AD based on the known literature in the occupational field. The outcome shows that all these concerning elements could get attributed to Hg. However, recent studies did not investigate the molecular level of Hg exposure in AD. The present review highlights the interactions between Hg and AD in neuronal degenerations, apoptosis, autophagy, oxidative stress (OS), mitochondrial malfunctions, gastrointestinal (GI) microflora, infertility and altering gene expression.

## 1. Introduction

Hg was used several thousand years ago by Chinese people to prepare red ink from cinnabar [1]. In addition, Hg was found in Egyptian tombs used as a preservative or to ward off evil spirits. In ancient times it was believed that Hg prolonged life and maintained health, in contrast with the current state of knowledge regarding the properties of this element.

From the beginning of the 19th century, Hg was used to produce amalgams with other metals such as silver and gold. Furthermore, methylmercury (MeHg) and ethylmercury (EtHg) are still being used in various vaccines [2]. Nowadays, Hg is considered one of the most toxic widespread environmental pollutants, which has multiple effects on organisms even at low concentrations [3,4,5].

Exposure to Hg can occur from both natural and artificial sources. Hg can reach ecosystems through anthropogenic activities, including the burning of fossil fuels, Chlor-alkali industries, mining, and the use of coal and petroleum, which can result in Hg exposure. The natural sources for this element are volcanic activity, erosion, the volatilization of Hg present in the marine environment, forest fires and biomass burning [6,7].

Minamata City is internationally known for “Minamata disease”, a neurological disorder caused by MeHg ingestion from contaminated food. This was an ecological catastrophe and a massive Hg poisoning caused by irresponsible industrial chemical disposal in the Minamata Gulf. The route of exposure to MeHg was through the consumption of contaminated fish and shellfish, the primary food sources of the local population. The disease symptoms include uncontrolled limb movements, impaired motor functions, impaired speech, disturbed vision and hearing [8,9,10]. The World Health Organization (WHO) [11] deemed Hg to be one of the top ten chemicals or groups of chemicals of public health concern. In 2013, 128 countries signed Minamata Convention to protect the environment and human health from anthropogenic emissions and releases of Hg and Hg compounds [12,13].

Hg has possible toxic effects on the nervous, digestive and immune systems and the lungs, kidneys, skin and eyes [11,14]. The route of entry of a noxious substance like Hg can be cutaneous, digestive or inhaled [15]. In case of severe intoxication with Hg, it can lead to death [16]. Scientists investigated the neurological toxicity of Hg compounds in different species, particularly in mammals, fish, birds and reptiles. Through maternal milk, exposure can lead to OS in the cerebellum of weanling mice [17]. In mammals, Hg-induced pathogenesis of the nervous system, increased membrane permeability and neuronal protein production lead to disruptions of metabolic functions and structures, causing a loss of enzyme functions, the difficulty of locomotion, reduced vision, general weakness, tremors, loss of consciousness and ultimately death [18]. According to Chakraborty [4], there has been an increasing number of dementia cases worldwide. The connection between increasing Hg exposure and neurodegenerative disorder is yet unknown.

## 2. Methodology

The literature databases searched for information used in the present manuscript until inception (January 2022) were: ScienceDirect, PubMed/Medline, ISI Web of Knowledge, Scopus and Cochrane Database of Systematic Reviews (CDSR) databases for publications.

Several keywords such as “mercury”, “Alzheimer’s disease”, “oxidative stress”, “apoptosis”, “autophagy”, “gut microbiota”, “fertility” and “infertility” were used during the database search. We completed the selection of all the relevant literature based on title, abstract information and full content.

Conference posters, letters to the Editor, preprints or computational simulations have not been considered suitable. We performed analysis only on the English-written articles. Five authors (E.P.; D.I.; V.R.; A.R.; O.-D.I.) independently inquired about the information, and any existing differences were solved, by common consent, with the remaining four authors (R.J.; A.C.; M.N.; B.D).

## 3. Results

### 3.1. Hg Availability

There are three forms of Hg, including elemental Hg (Hg^0^**;** metallic), inorganic (Hg^2+^) and organic Hg compounds such as MeHg [19].

#### 3.1.1. Hg^0^

Hg^0^, also known as quicksilver, is liquid at room temperature and is used in thermometers, dental amalgams, fluorescent light bulbs, mining and some industrial processes. During the burning of coal and other fossil fuels, Hg^0^ gets released into the air [20,21].

Hg^0^ is a particularly volatile liquid at 25 °C temperature, with a vapour pressure of 0.00185 mm. The volatilization of Hg is directly proportional to temperature; the higher the temperature, the higher the amount of Hg in the air. If the Hg vapour exceeds 0.05 mg·m^−3^ of air, it is a chronic exposure to Hg that results in cumulative poisoning. In humans, absorption of volatile Hg is 70% to 80% through the lungs and 3% dermally. If ingested, Hg^0^ is absorbed slowly and passes through the digestive system without causing damage [22]. Its solubility in lipids makes it more easily absorbed by the body, thus reaching the circulating alveoli and penetrating the blood–brain barrier (BBB) to the CNS. Hg^0^ has a relatively long period of remanence in the brain, and it can be detected even a few years after exposure [22].

#### 3.1.2. Hg^2+^

Hg^2+^ compounds develop when the Hg combines with other elements, such as sulphur (S) or oxygen (O_2_), to form compounds or salts. Naturally occurring Hg^2+^ compounds are used in several industrial processes, including the manufacture of other chemicals [18]. Following Hg^2+^ ingestion, the GI tract absorbs between 7% to 15% of the doses. Some skin products and ointments contain Hg^2+^ salts; one of the many possibilities of Hg^2+^ intoxication is that these toxicants are absorbed dermally. Inorganic salts of Hg are not soluble in lipids; they do not penetrate the BBB easily and are excreted in the faeces and urine in an estimated 60 days [1]. Hg is biomagnified in the food chain and induces various toxic effects in the organisms from the aquatic systems [18]. In fish, exposure to Hg^2+^ can lead to deterioration of motor skills and alterations in anxiety responses and modify thyroid hormone levels and gene expression in the hypothalamic-pituitary-thyroid (HPT) axis [23,24].

Hg^2+^ can cause brain damage by changing calcium (Ca) homeostasis via calcium adenosine triphosphate (Ca-ATP) pump impairments, can obstruct the assembly process and lead to tubule disintegration through attaching to the thiol groups of alfa (α)-tubulin and beta (β)-tubulin, the primary monomeric protein part of the neuronal microtubules. Furthermore, Hg^2+^ can affect the CNS by repressing glutamate uptake, thus expanding its release in the extracellular space [25].

#### 3.1.3. Organic Hg

Organic Hg compounds assemble when the Hg combines with carbon (C). Microscopic organisms in water and soil can convert Hg^0^ and Hg^2+^ into an organic Hg compound, MeHg, which accumulates in the food chain. Thimerosal and phenylmercuric acetate represent other organic Hg compound types used as preservatives in small amounts. All forms of Hg are toxic, but studies show that the organic forms of Hg are generally more toxic to organisms than the inorganic forms [26].

MeHg is a well-known organic compound of Hg, naturally produced in the aquatic environment by aquatic microorganisms through the methylation of Hg^2+^. Under normal potential of hydrogen (pH) and temperature conditions, microorganisms from the aquatic environment can transform Hg^2+^ into organic Hg (mainly MeHg) and vice versa. The brain represents the primary target of MeHg exposure. The organic methylated Hg compound conveniently passes the BBB and is considered one of the most neurotoxic forms of Hg [2,16,18,21].

### 3.2. Toxicological Effects of Hg

Several toxic effects of Hg ions are corrosive action, enzyme inhibition and protein precipitation; in addition to the sulfhydryl Hg groups, they bind to phosphoryl (-PO_3_^−^), amide (R-N-R), amine (-NH_2_) and carboxyl (-COOH), and with these groups formed, proteins are readily available but sensitive to reaction with Hg. Thus, Hg creates an irreversible bond with an enzyme, which changes its conformation and prevents it from adhering to its substrate [22].

**Figure 1:**
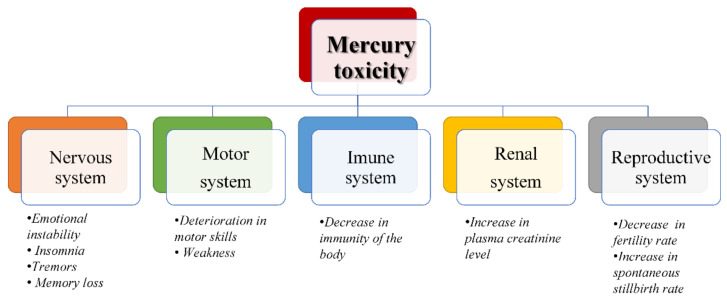
Symptoms associated with Hg poisoning of organ systems (data modified from Zahir et al. [3]).

Over 250 symptoms are associated with Hg exposure (Figure 1), and they can tangle accurate diagnosis. Medical diagnosis starts with a physical examination and patient history. In humans, laboratory analysis consists of blood, urine, hair analysis and, if necessary, a tissue biopsy. Hg gets rapidly removed from the blood system, segregated and redistributed to different tissues and, in this state, a correlation between the concentration of Hg in the blood and gravity of Hg poisoning cannot be made. Once in the body, Hg immediately finds its way through the brain, ganglia, spinal cord, peripheral neurons and autonomic ganglia to which it attaches tightly. The CNS is the one that takes care of storing Hg; the transient and residual distribution of Hg in systems can cause a large number of symptoms in different organs [27].

#### Clinical Studies on Toxicological Effects of Hg

Hg plays a crucial role in many health problems with harmful consequences, primarily targeting the brain and other components of the CNS [28,29,30].

According to da Rosa-Silva et al. [31], MeHg exposure creates an imbalance in redox reactions in the occipital cortex and the liver and generates hepatic glycogen accumulation and neurodegeneration. Organic Hg can affect the cerebellum and lead to impairments such as acute psychosis and erethism, apoptosis, neuropsychological disorders, oxidative damage, neuroblastomas and glioblastomas [32,33].

In recent decades, authors have been using zebrafish (*Danio rerio*) in research studies (Table 1) to the detriment of rats or mice due to their small size, high degree of similarity to the human genome, low costs, visual accessibility and accelerated embryo development. In conclusion, researchers prefer to use zebrafish larvae and embryos in experiments [34].

In 2019, in a compelling study by Zhu et al. [42], at a concentration of 0–400 nM HgCl_2_ and after 96 h of exposure, zebrafish exhibited a decreased survival rate, shrinkage in body length and eye diameter, delayed hatching and reduced locomotor activity. Cruz et al. [37] observed only 24 h after exposure that the larvae exposed to 20 μg·L^−1^ of HgCl_2_ showed a decreased CAT activity and increased MDA levels.

Scientists have increasingly been using zebrafish embryos for their transparency, thus effortlessly monitoring their changes. By exposing the zebrafish embryos to a concentration of 5–1000 μg·L^−1^ MeHg, Hassan et al. [36] discovered it to induce mortality. In addition, Zhang et al. [5] and Kusik et al. [39] witnessed this effect 1–7 days after exposure to a concentration of 1–16 μg·L^−1^ HgCl_2_.

The skeletal malformation is a general symptom of Hg exposure; Abbott et al. [38] observed this effect 24 h after exposure of zebrafish embryos to a concentration of 100–1000 μg·L^−1^ MeHg, while Dong et al. [44] noted that, in the case of medaka fish exposed to a concentration of 0.001–10 μM CH_3_HgCl, skeletal malformations and pericardial oedema appear after ten days.

Clinical studies on Hg exposure revealed OS and memory loss presence in adult zebrafish, these being among the main effects that induce non-degenerative diseases [40,45].

In studies with a duration of 45 days (Table 2), at a dose of 375 μg·kg^−1^ per day HgCl_2_, Aragão et al. [46] and Teixeira et al. [47] observed motor and cognitive dysfunctions, apoptosis and OS in 90 days old male Wistar rats. In addition, in 2014, Teixeira et al. [48] conducted a study this time on 150 days old male Wistar rats and witnessed short and long-term memory impairments and accumulation in the hypothalamus and cortex.

Reactive oxygen species (ROS) are the leading cause of many neurodegenerative diseases (for example, AD, Parkinson’s disease-PD), exposure to Hg, especially HgCl_2_ favouring their production [46,53,55,57].

Fujimura et al. [61,62] examined male C57BL/6NJcl mice, less than six weeks old, and, after eight weeks of MeHg administration of 30,000 µg·kg^−1^, noted neuronal degeneration and reduced antioxidant enzymes expression (manganese superoxide dismutase—MnSOD and glutathione peroxidase—GPx1).

After exposure to Hg vapour for 45 days at 9 h per day at a concentration of 1 mg·m^−3^, Altunkaynak et al. [60] studied adult female white Wistar rats (8–10 weeks old) and detected the loss of Purkinje cells, deleterious effects of the cerebellum structure, while in female Sprague-Dawley rats (61–67 weeks old), at an exposure of fewer than 11 days for two hours per day, but at a higher concentration of 2–4 mg·m^−3^, Goering et al. [51] observed ROS increase in brain and kidney.

Administration of a higher amount of HgCl_2_ (over 1000 µg·kg^−1^ per day) can lead to fibrosis and asthma [53] or even to histopathological changes in the lung tissue [48]. In 2019, Oliveira et al. [58] remarked that the first dose of 4.6 µg·kg^−1^ and subsequent doses of 0.07 µg·kg^−1^ per day of HgCl_2_ generate anxiety and memory impairment in rats after 30 days.

### 3.3. Hg Poisoning Implications in AD

#### 3.3.1. Causes of AD

AD is an irreversible, progressive brain disorder, which affects memory and cognitive function. AD is the most common cause of dementia among adults [64,65,66].

In most people with AD, symptoms appear in the elderly. This process involves two proteins called β-amyloid (senile plaques) and tau (neurofibrillary tangles), which become toxic to the brain [67]. It seems that abnormal tau aggregates, ultimately forming tangles inside neurons and β-amyloid, cluster into plaques, gently building up between neurons. As the level of amyloid reaches a tipping point, there is a rapid spread of tau throughout the brain. Abnormal deposits of proteins form amyloid plaques and tau tangles through the brain. Once healthy neurons stop working, they lose connections with other neurons and eventually die. Hg, known as one of the most toxic heavy metals, is frequently believed to set in motion AD [4,65].

Researchers focused on investigating interactions with and between pathways thought to be involved in neuronal degeneration, such as inflammation, malfunctioning mitochondrial mechanisms, apoptosis, autophagy and OS, for a better understanding of the neurodegenerative process and its workings [25].

##### OS

**Figure 2:**
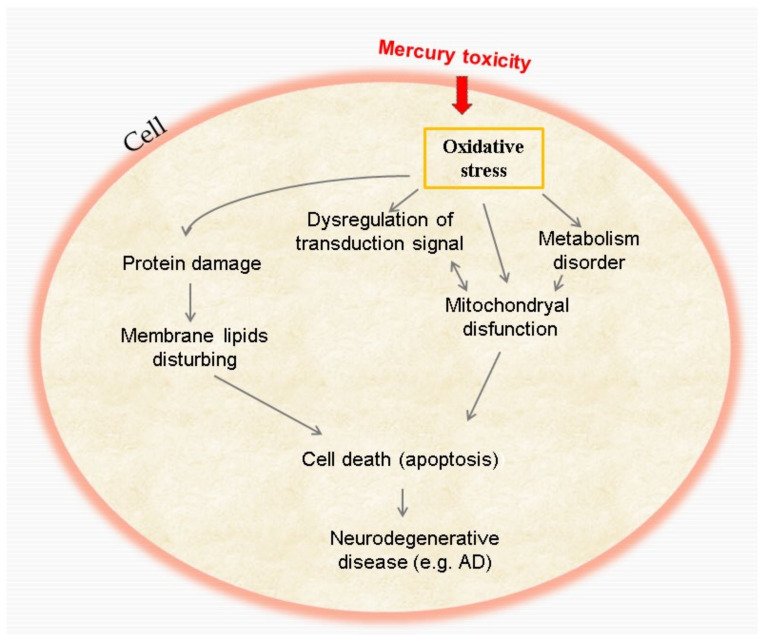
Mechanism of Hg toxicity and its effects at the cellular level.

OS is the process of a quantitative imbalance in the production of ROS and antioxidants, leading to cell damage (Figure 2), protein oxidation or the appearance of various diseases, such as neurodegenerative diseases (AD or PD). ROS attack proteins, oxidizing their base structure and side groups [68,69].

OS plays a decisive role in AD onset and progression by amplifying key events [70].

Hg stimulates the formation of insoluble β-amyloid, which plays a crucial role in the pathogenesis of AD and causes OS and neurotoxicity in vitro. Thus, AD, characterized by the destruction of neurons is associated with the aggregation of β-amyloid protein in the form of amyloid plaques. Accumulation of β-amyloid peptide leads to OS and mitochondrial dysfunction before plaque pathology [71].

The chain transport of mitochondrial electrons to the cytochrome oxidase complex consumes approximately 98% of molecular O_2_. The rest of the O_2_ gets reduced to hydrogen peroxide (H_2_O_2_) and the superoxide radical (O_2_^−^). Under stress and during ageing, the electron transport system can increase considerably, leading to ROS forming. Thus, mitochondria are both a source and a target of toxic ROS. Mitochondrial dysfunction and the production of ROS may play a significant role in the pathology of AD (Figure 2) [72].

Experimental models suggest that OS plays a crucial role in the toxicodynamic of heavy metals, including Hg [73]. Both in vivo and in vitro models show that Hg exposure can cause OS in the biological system [74,75,76,77,78] with the generation of ROS, glutathione (GSH) depletion and decreased sulfhydryl (–SH) protein group [79].

The CNS is the main target of MeHg toxicity, reflecting its efficient transport in the brain. Scientists examined the mechanism of how MeHg crosses the BBB in rats, as well as its absorption by neuronal cells [79,80,81].

Petroni et al. [82] demonstrated that harmful effects of MeHg ameliorate through the use of an antioxidant, N-acetylcysteine (NAC), and a calpain inhibitor, N-[N-[(Phenylmethoxy) carbonyl]-L-valyl] -phenylalanine (MDL-28170). NAC was cytoprotective and reduced phosphorylation but also the production of ROS.

The brain is composed primarily of easily oxidizable lipids, has a high O_2_ consumption rate and has no substantial antioxidant defences, thus becoming vulnerable to oxidative damage [83]. In 2018, Liguori et al. [84] showed a higher level of oxidation in the brain with ageing, which is the most consistent risk factor for AD.

Exposure to Hg in low concentrations induces OS, cellular cytotoxicity and an increase in β-amyloid, associated with neurodegenerative disorders such as AD in adults [85].

##### Apoptosis

Apoptosis is a natural form of programmed cell death (PCD), a process by which cells trigger their self-destruction in response to a particular signal. The defective development of apoptosis has a special significance in the pathogenesis of some diseases. The disorder in apoptosis processes leads to a series of pathological conditions by intensification or reduction. Excessive apoptosis can contribute to neurodegenerative diseases like multiple sclerosis, AD or PD [86].

To establish the correlation between neurodegenerative disease and heavy metals, Lee and his colleagues oversaw a study of 130 people and a group of 80 AD patients. The experts applied the T-test for blood and serum analysis. The test results showed no statistically significant values or correlation between AD and heavy metals, mainly Hg [87].

According to Watanabe et al. [88], after four hours of exposure of cells to 0.1 µM, 1 µM MeHg, results in deoxyribonucleic acid (DNA) laddering, generated by inter-nucleosomal DNA fragmentation, exhibit a delay in cell death, and starting with 0.5–1µM, MeHg induces cell death. This study shows that Hg, even at low concentrations, induced delayed caspase activation, eventually leading to cell death. Hg, through its effects, inhibits the progression of the cell cycle mitosis and generates acute necrosis and delayed apoptosis by blocking cell expansion in different cell types. Well-known essential mediators of cell survival, differentiation and apoptosis are ROS [88]. 

Some studies show that OS can cause apoptosis through the mitochondria-dependent and mitochondria-independent pathways. Mitochondria play a significant role in some cellular processes, including the initiation of apoptosis [25,89]. Scientists detected mitochondrial dysfunctions in rare monogenic mitochondrial diseases and various common pathological conditions, such as neurodegenerative diseases [25]. 

Researchers have found proof of a correlation between AD and Hg exposure. Studies have shown that deceased patients who suffered from neurodegenerative diseases have higher levels of Hg in their nerve tissue than healthy people. According to Cariccio et al. [25], neurotoxic proprieties of MeHg can alter messenger ribonucleic acid (mRNA) expression, decline mitochondrial function, increase ROS productions and induce apoptosis. Mitochondria is the primary site for ROS generation under neurotoxic proprieties of OS, ROS aggregation and MeHg [89,90].

##### Autophagy

Autophagy received increasing importance because scientists believe it plays a decisive role in the pathological genesis of neurodegenerative diseases (PD, AD) [91,92,93,94], where impaired exclusion of atypical and harmful protein clusters initiates the cell’s decline and even destruction [94]. Autophagy indicates the cellular recycling mechanism where cytoplasmic constituents get sent to lysosomes for discarding, thus supporting homeostasis at the cellular level by safeguarding cells from useless cellular residues [92,94,95,96].

Recent studies describe three significant varieties in autophagy: macroautophagy, microautophagy and chaperone-mediated autophagy. Macroautophagy is represented by the creation of the autophagosome, an uncommon double-membrane cellular structure, while in microautophagy, lysosomes dispose of cytoplasmic residues through internal invagination of their cell membrane and, in the case of chaperone-mediated autophagy, cytosolic chaperone conveys protein clusters to the lysosomal membrane where they are incorporated [92,96].

Persistent dysfunction of the autophagic system leads to a build-up of unprocessed degraded components and failure to process them, weakening the autophagosome surface and generating toxicity, a process noticed in AD [96]. Scientists tried to use the acceleration of the autophagic pathway as a treatment on AD animal models and got conclusive results for two “autophagy upregulators”, trehalose and rapamycin [97].

Analysis of brain tissue sections from human subjects with neurological decline displays the accumulation of autophagic vacuoles and, further test results revealed that AD patients have elevated autophagosome numbers in their brains [94,98]. Experts witnessed, in several AD studies, an escalation in OS and the development of ROS, thus resulting in an increased autophagic action, which induces cellular destruction [98]. Researchers advanced the idea that robust induction of autophagy may initiate a cellular death process in excitotoxic induced neuronal decline [94]. Interestingly, autophagy influences the neurological toxicity caused by exposure to essential and non-essential trace elements such as manganese (Mn), copper (Cu), cadmium (Cd), lead (Pb) and MeHg [94].

After chronically subjecting human neural stem cells to MeHg (0.01–1 µM) during an in vivo experiment, Chang and his colleagues [99] noted a dose-related correlation with low levels of mammalian target of rapamycin (mTOR) protein—a negative regulator of the autophagic cellular action [94,100], thus transforming the beneficial process of autophagy in a degenerative mechanism [94].

Yuntao et al. [101] administered MeHg to rat astrocytes and, after dosing, examined possible biomarkers of autophagy and observed that the astrocytes accumulated autophagosomes and had elevated levels of Beclin-1 protein, thus upregulating the autophagy pathway. The experts obtained cytoprotection by increasing autophagy with rapamycin administration and cytotoxicity from overriding autophagy with chloroquine dosing. In addition, the authors demonstrated that, by lowering the cellular apoptosis process, autophagy exerts a protective influence and OS sets in motion the autophagic mechanism.

#### 3.3.2. Does Hg Poisoning Induce Symptoms for AD?

Researchers believe that the degenerative AD brain absorbs or accumulates more easily Hg. Between 3% to 5% of cases likely have a genetic origin. On the other hand, scientists consider environmental factors one of the fundamental promoting causes of the onset and progression of AD. Countries with a flourishing industry and energy production have the highest number of cases of dementia due to increasing Hg emission. Various authors associate Hg with AD since countries with the highest Hg emissions have an increased number of AD cases. China has the highest number of patients with dementia, 19.9% of cases, followed by the United States with 8.9% cases, India with 8.7% cases, with an estimated 46.8 million cases worldwide. Researchers anticipate an increase in the number of cases of patients with dementia in industrialized countries such as China and India; by 2030, scientists estimate that the number will double, and by 2050, the number will triple [4]. The severity of AD in brain regions correlates with the number of neurofibrillary tangles. In some cases, before the onset of clinical symptoms, the changes in fibrillary nerve cells begin up to 50 years earlier. This aspect would exclude age as a cause. Population between 20 to 30 years with no clinical symptoms may have a low number of neurofibrillary tangles. By the age of 80, 90% of individuals display neurofibrillary tangles in the brain [66].

Numerous studies highlight the role of Hg as an extensive factor in the pathological effects of AD [20,102]. Epidemiological studies made a comparison between patients with AD and healthy subjects. Investigations have shown that patients with AD have a higher level of Hg blood concentration in cerebral tissue; the level of Hg was twice as high as that of depressed patients and patients without psychiatric disorders [103]. Researchers assert that chronic exposure to Hg can get misdiagnosed as AD because of the symptoms that include personality changes and memory loss in aged people. Nonetheless, meta-studies sustain a potential connection linking Hg and AD [66].

Exposure to one of the three forms of Hg has a drastic effect on the body. Table 3 shows the main symptoms identified a while after Hg exposure. In case of injection or ingestion of metallic Hg, the repercussions on the body are immediate. Symptoms of exposure to Hg vapour appear even a few years after exposure and in the case of liquid Hg in the first two weeks.

Yildirim et al. [106] present the case of a family with five members, aged between 20 and 54 years, exposed to liquid Hg through dermal contact and or inhalation. Thus, in case 1, the 54-year-old woman suffered the lengthiest exposure, the clinical presentation being severe and death, and in the case of the other four people, the clinical presentation was less significant because of lower dose exposure.

In 2010, Feitosa-Santana et al. [110] observed a correlation between the exposure time to Hg vapours and the visual system, prolonged exposure to Hg vapours correlating with visual impairments.

Inorganic salt poisoning is well known, but few reports of oral intoxication with metallic Hg exist, as its GI absorption is poor and of no concern. Katsuma et al. [108] describe a case with ingestion of metallic Hg, observing severe pneumonitis, anuria and a high concentration of Hg in the blood, 1577 μg·L^−1^. In addition, de Souza et al. found a concentration of 1929 μg·L^−1^ in the blood of a 46-year-old woman, after exposure to the same form of Hg for two years, symptoms including severe pain, ischemia, erythematous lesions and cyanosis of the left hand [113].

Vahabzadeh et al. observed in three cases of exposure to metallic Hg, that Hg concentrations in the urine differ depending on age and time of exposure. In the first case of a 30-year-old man, exposed eight hours per day, for a period of 50 days, the concentration of Hg in the urine was 760 μg·L^−1^, in the second case of a woman of 53 years, exposed for the same time, presented 326 μg·L^−1^ Hg in urine, and in the third case of a 20-year-old man, the exposure was higher than in the first two cases, 60 days, 8 h per day, the concentration of Hg in the urine being close to the first case, 635 μg·L^−1^ [109].

Metallic Hg injection, often used as a method of suicide, is regularly observed among young people; noticed main effects are weakness, chest pain, arthro-myalgia in the antecubital fossa, schizophrenia and inflammatory soft tissue lesions [104,107,111,112].

Sarikaya et al. [105] cite a case of a 36-year-old woman exposed to metallic Hg after her daughter brought it from school; after exposure, the woman’s other daughter (14 months old) died before being hospitalized, but, after NAC administration, the woman managed to escape without sequelae.

### 3.4. The Gut Microbiota as a Modulator of Hg Neurotoxicity

Commensal enteric microorganisms ensure the host’s eubiosis and prevent impairment of the intestinal barrier. Their dysfunction causes a leaky gut marked by elevated circulating lipopolysaccharides (LPS) that cross the BBB. Consequently, the brain returns signals through which the immune system is activated and subsequently triggers a pro-inflammatory cascade of cytokines.

Two recent studies of Ruan [114] and Zhao et al. [115] support the above argument. They systematically evaluated the toxicological effect on sixty Kunming mice of the HgCl_2_, Cu, or mixture in different doses: the first sign noted was reduced weight three days after the initial exposure (*p* < 0.01). Further proof that suggests the existence of a pathological condition is the level differences of oxidative markers. Specifically, a disruption of the antioxidant defence (superoxide dismutase-SOD, *p* < 0.01; GSH, *p* < 0.01), along with lipid peroxidation marker above the optimal status (MDA, *p* < 0.01). The *16S* rRNA analysis of cecum tissues on the 90th day pointed out a shifted microflora.

Zhao et al. [116] reproduced the above protocols by combining the number of experimental models [115] and chronic exposure [114]. They demonstrated overexpression of five pro-apoptotic genes, among which were c-Jun N-terminal kinase (*Jnk*), apoptosis signal-regulating kinase 1 (*Ask1*), apoptosis regulator BAX (*Bax*), caspase 3 and tumour necrosis factor alphaα (*TnfA*), in parallel with a significant decrease of B-cell lymphoma 2 (*Bcl2*). 

A group of scientists tested the working hypothesis that the toxicological profile of Hg sulfide (HgS) is not the same as MeHg and HgCl_2_. Zhang et al. [117] orally administered four different doses in Kunming mice for one week - HgS (30 mg·kg^−1^), MeHg (3.1 mg·kg^−1^), HgCl_2_ (33.6 mg·kg^−1^) and Zuotai (30 mg·kg^−1^). Operational taxonomic unit results show that *Firmicutes* and *Proteobacteria* were prevalent in HgS treated mice. *Bacteroidetes* and *Cyanobacteria* predominated, and *Firmicutes* decreased in the group of mice treated with HgCl_2_. Researchers noted increased *Rikenellaceae*, *Lactobacillaceae*, *Helicobacteraceae*, *Odoribacteraceae*, *Porphyromonadaceae* in HgS and HgCl_2_ treated mice and reduced *Prevotellaceae* and *Lactobacillaceae*. HgS and Zuotai exercise differential effects on gut microflora compared with MeHg and HgCl_2_. There was a high accumulation of Hg in the duodenum and ileum after MeHg and HgCl_2_. On the other hand, MeHg had a subtle effect by comparison with HgS and Zuotai that had no effect.

Jiang et al. [118] highlighted that an oral intake of *Lactobacillus* (*L. brevis* 23017-10^9^ CFU/200 μL) promotes short-chain fatty acids (SCFAs) generation and precludes weight loss; thus associating depletion with neurodegeneration [119]. Apolipoprotein E (APOE4) human carriers faeces keep a low abundance of *Ruminococcacee*, entities needed in the fermentation of SCFAs [120]. Species from *Cloacibacterium*, *Rhodobacter* and *Acinetobacter* [121,122] are well-known SCFAs generators. These communities increase significantly in fish exposed to MeHg [123]. MeHg in fish promotes several modifications of taxa involved in xenobiotic metabolism and metal removal. 

*Lactobacillus casei* BL23 and *Lactobacillus acidophilus* ATCC4356 are lactic bacteria widely utilized in current methodologies due to their ability to diminish the permeability driven by both inorganic and organic MeHg (20–94%) through NCM460/HT29-MTX monolayers [124]. Whilst deposition in the liver and kidney was inconsequential, Lin et al. [125] contradict these findings. Primarily favourable proliferative conditions of pathogenic species such as *Bacillus* are being created [125]. *L. brevis* 23017 grants protection against Hg toxicity by protecting the small intestine villi and maintaining the integrity of the mucosal barrier via modulation of the tight junction proteins [117].

*Lactobacillus acidophilus*, *Lactobacillus fermentum*, *Bifidobacterium lactis*, *Bifidobacterium longum* and SLAB51 are powerful vehicles in restoring the cognitive activity in Aβ1-42-injected rats, activating class III deacetylase histone (SIRT 1); thus, adequate doses normalize the redox imbalance and enhance the learning process and memory [126]. Contrarily, they increase the MDA level (*p* < 0.0001; *p* < 0.05) as well as the SOD activity (*p* < 0.05) [127].

MeHg provoked pronounced alterations in the metabolism of various other model organisms because it binds covalently to the cysteine residues and inhibits the growth of *Lactobacillus* [128]. Fatty acids (oleic, stearic and palmitic acids) concentration fluctuates concomitantly with an elevated amount of glycerol. *Bacteroidaceae* and *Desulfovibrionales* increased several folds—2 and 7.5 in MeHg-treated rats. The presence of these species negatively correlated with pyroglutamic, aspartic, xanthurenic acid and glycine concentrations [129,130]. From a molecular point of view, it stimulated fluctuations in glutamate, brain-derived neurotrophic factor (BDNF), gamma-aminobutyric acid (GABA), dopamine (DA) and tryptophan (Trp).

Studies carried out in vitro and in vivo led to intriguing findings. Those in vitro revealed that incubation of MeHg with experimental models or human stool results in the production of Hg^0^. In vivo protocols affirmed the significance of bacterial demethylation. This process plays a fundamental role in the successful removal of Hg. In cases of suppression or absence, it lowers the excretion of Hg [131]. *Peptococcaceae* possess the ability to demethylate MeHg and to increase faecal excretion in rats [130]. There are two-genes clusters behind this *HgcAB* mechanism that these microorganisms require for Hg methylation [132]. Several *HgcAB*-encoding strains affect the methylation potential [117,133]. Siderophores bind to Hg and function as chelators to improve the excretion in the faeces using the modulation of iron (Fe) carriers amongst other vital functions [134]. Thus ensuring the control of metal bioavailability and brain-related trophic nutrients that prevent bacterial overgrowth [135].

### 3.5. Hg’s Associations with Fertility Potential

In retrospect, the critical situation in Minamata City between 1950 and 1974 was one of the most regrettable events. There was a slight increase in spontaneous stillbirths, especially among male infants and a decrease in artificial stillbirths after severe MeHg poisoning in this Kyushu municipality and neighbouring municipalities. The crude fertility rate was low and significantly lower compared to Kumamoto Prefecture (*p* < 0.001) [136].

Consumption of seafood contaminated with trace elements could be behind the positive correlation of the specific markers. Non-linear higher Hg in ovaries and a lower probability of biochemical pregnancy and live birth (*p* = 0.05) (weekly–60% versus annually 16%), semen parameters (50%, 46% and 31%) and reproductive outcomes in both genders using hair or urine Hg levels (0.72 ppm/0.3–0.6) suggest primary sources of exposure [137,138,139,140].

Arsenic (As) and Hg were not significantly correlated with semen parameters of occupationally exposed men [141] and decreased by up to 31% in the first 12 months after the bariatric surgery in women of reproductive age [142]. Although there is no direct association between Hg and infertility, the current evidence is controversial. Univariate and multivariate analyses showed no significant linear relationship between blood Hg and infertility [143].

While the effect of acute exposure lasted less than a week in zebrafish embryos, Hg^2+^ disrupted gametogenesis and sex hormones balance at early stages, which transmitted to a lesser extent to F1 female offspring. The scientists found no significant intergenerational peculiarities in F1 males and F2 females [144]. The Hg in the blood of rats is comparable to that of individuals characterised by high GPx1/4 levels [145].

Omega−3 fatty acid (n3PUFA) diet was 0.60 ppm, and the probability of mature oocytes was inversely correlated with hair Hg concentrations (RR = 0.81) and positively correlated with antral follicle count (AFC) in women [146,147]. Hg in the hair and fish consumption correlates directly by a factor of 70 with maternal age, education and the risk of never getting pregnant and inversely with pre-pregnancy body mass index (BMI) [148,149,150]. Curcumin and Se promote neurodevelopment, alleviate anxiety and neutralise the perinatal effect of Hg but may affect menstrual cycle length (MCL) [151,152]. These compounds increase the expression of neurotransmitters such as DA, serotonin (5-HT), acetylcholinesterase (AChE) and GSH [153,154].

## 4. Hg-Mechanisms of Action

According to Olivieri et al. [155], Hg induces OS [156] and Aβ production. Studies have indicated that Aβ accumulation in the brain is a hallmark of AD pathology [157].

Hg exposure disrupts ℽ-secretase activity and leads to an increase in Aβ levels and OS through free radical production, which mediates Aβ toxicity [158]. In some patient cases of AD, researchers reported a DNA amino acid substitution in amyloid precursor protein (APP). The APP processing pathways are altered by the ℽ-secretase modulators complex, leading to increased synthesis of the neurotoxic protein Aβ (1–42) [159]. In the brain and in vitro, the activity of the kinase creatine (C) protein, in particular, is reduced by Hg in a concentration-dependent manner. In recent assessments, Hg concentration inhibits the binding between phorbol ester and kinase C protein [155]. 

Hg levels may be responsible for the α-secretase activity by protein kinase reduction, leading to increased Aβ formation due to activation of the α-secretase pathways by kinase C protein [155]. 

An additional pathological feature of AD is the presence of neurofibrillary tangles and their elements, consisting mainly of hyperphosphorylated tau. The phosphorylation state of tau protein can be significantly affected by Aβ and OS [155]. 

Studies have shown that OS cause cell death via both pathways, mitochondria-dependent and mitochondria-independent, primarily by accumulated, produced and impaired functionality in the mitochondria. After production, O_2_^−^ is immediately converted to H_2_O_2_ in the mitochondria by Mn-SOD. The H_2_O_2_ quickly reaches the cell membranes and behaves like a redox signal from the mitochondria to the cell [25].

Hg reaches the motor cortex and accumulates in deposits, increasing nitric oxide generation, promoting lipid peroxidation and disrupting membrane lipids. These facts lead to an apoptosis process that kills glia and neurons [48].

Therefore, Hg toxicity can cause OS, increase APP expression on neuron and amyloidogenic pathways, suppress kinase C and stimulate Tau hyperphosphorylation [160].

## 5. Protective Compounds against Hg-Induced Neurotoxicity

Presently, knowledge about available treatments and products that can protect or mitigate the effects in Hg-exposed individuals is insufficient, although we understand that Hg exposure can affect brain development and function.

Lately, several studies have established that natural products with antioxidant and free radical scavenging properties can ameliorate or protect against the brain effects induced by diverse forms of Hg (Table 4). For example, Owoeye et al. [161] have shown that the aqueous extract of *Celosia argentea* and vitamin E can attenuate the oxidative and histological changes induced by HgCl_2_ exposure in rats. Owoeye and his collaborators proved, during a previous study, that pretreatment with an extract of *Launaea taraxacifolia* significantly improved the histological changes in the rat brain and reduced the effects of HgCl_2_ on haematological and behavioural parameters [162].

Several Hg toxicity experiments investigated whether curcumin is a biologically active compound [164,165,173,174]. Agarwal et al. observed that pretreatment with curcumin had a prophylactic effect on Hg-induced OS parameters, such as lipid peroxidation and GSH levels, as well as the activities of SOD, GPx and CAT [164]. In addition, curcumin may have a protective effect when administrated to the offspring during the perinatal period by attenuating the behavioural and biochemical changes induced by HgCl_2_ exposure [165].

Using an extract of *Dendropanax morbifera Léveille* leaf as a treatment significantly reduced Hg concentrations in hippocampal homogenates and increased antioxidant enzyme activities. Sumathi et al. showed that *Bacopa monniera* potentially protects the brain from oxidative damage caused by MeHg-induced neurotoxicity in rats [168]. Pretreatment of rats with DAS inhibited an increase in lipid peroxidation, elevated (AChE) and GSH levels and caused a decrease in *TnfA* level, which was higher in the Hg-treated group [166].

Some studies found that selenium (Se) supplementation reduced Hg-induced neurotoxicity [20,171]. For example, El Asar et al. demonstrated the essential role of Se in inhibiting the mitochondrial apoptotic pathway via modulation of the apoptotic markers *Bax*, *Bcl2* and Caspase-3 [171]. On the other hand, co-administration of NAC can attenuate the Hg toxicity in the perinatal brain [20]. NAC treatment prevented the reduction in DNA synthesis and the marked increase in caspase-3 immunoreactivity.

## 6. Conclusions

It is indisputable that Hg plays a vital part in neurotransmitter metabolism, the progression of neuroinflammation and AD evolution. Along with other heavy metals, Hg is an instrumental cofactor for AD. Various tests have confirmed its role in neurotransmitter metabolism in addition to the progression of neuroinflammation, although other factors may also lead to the development of AD in distinct forms of AD. Currently, there is sufficient data to show that Hg can affect the assembly of tubulin microtubules in the CNS. Clinical trials have linked high Hg concentrations in nervous tissue and blood of patients with AD. Hg may be a risk factor for AD and the findings suggest that it deserves further investigation to understand its mechanisms. Some chemically synthesized and natural compounds can reduce and, maybe, attenuate Hg neurotoxicity, but most scientists conduct experiments on model organisms. At the moment, researchers are interested in minimising the effects of Hg on the human CNS.

## Figures and Tables

**Table 1:** Summary of some studies with neurological effects of Hg exposure.

Exposure Duration	Age of Exposure	Species	Chemical	Concentration	Major Endpoints	Reference
4–72 h (post fertilization)	embryos	zebrafish	MeHg	6 μg·L^−1^	Impaired development of the fin fold and the tail fin primordium, alterations in transgene expression	[35]
6–96 h (post fertilization)	embryos	zebrafish	MeHg	5–1000 μg·L^−1^	Delayed hatching, decreased cell proliferation rate within the neural tube, mortality	[36]
24 h	larvae	zebrafish	Mercury chloride (HgCl_2_)	20 μg·L^−1^	Decreased catalase (CAT) activity and increased malondialdehyde (MDA) levels	[37]
24 h	embryos	zebrafish	MeHg, HgCl_2_	100–1000 μg·L^−1^	Delayed toxicity, skeletal malformations, changes in morphology, severe morphological defects, mortality	[38]
24 h	embryos	zebrafish	HgCl_2_	1 μM		[39]
32 h	adults	zebrafish	HgCl_2_	1–15 μg·L^−1^	Memory loss, aggression, low swimming performances, OS	[40]
96 h	adults	zebrafish	HgCl_2_	7.7–38.5 μg·L^−1^	Degeneration, apoptosis	[41]
96 h	larvae	zebrafish	HgCl_2_	0–400 nM	A decreased survival rate, shrinking in body length and eye diameter, delayed hatching, reduced locomotor activity	[42]
7 days	embryos	zebrafish	HgCl_2_	1–16 μg·L^−1^	Delayed hatching and increased mortality	[5]
7−14 days	juvenile	White seabream	Hg^2+^	2 μg·L^−1^	Optic tectum cells reduction	[43]
10 days	embryos	Medaka fish	Methyl mercury chloride (CH_3_HgCl)	0.001–10 μM	Skeletal malformations, pericardial oedema	[44]
30 days	adults	Gilthead Seabream	MeHg	10 μg·L^−1^	OS accumulation in organs	[45]

**Table 2:** Summary of several studies used in this review to present the effects of Hg on rodents (g-grams).

Exposure Duration	Age of Exposure	Species	Chemical	Concentration	Major Endpoints	Reference
24 h	56 days old	BALB/c mice	MeHg	0–10,000 µg·kg^−1^	Accumulation in brain	[49]
5 days (pre-gestational), 19 days (pregnancy)	Adult	Female Wistar rats	MeHg	1000 µg·kg^−1^/day	Neuron loss in the brain, hippocampus and amygdala	[50]
11 days (2 h/day)	61–67 days old	Female Sprague-Dawley rats	Hg vapour Hg^0^	1; 2; 4 mg·m^−3^	ROS increase in brain and kidney	[51]
21 days	220–250 g	Male albino-Wistar rats	HgCl_2_	400; 800; 1600 µg·kg^−1^/day	Spatial memory impairments; hippocampal mitochondrial dysfunction	[52]
28 days	Adult	Male Wistar albino rats	HgCl_2_	1000 µg·kg^−1^/day	Histopathological changes in the lung tissue; OS	[53]
28 days	49 days old	Male Wistar rats	MeHg	0; 20; 200; 2000 µg·kg^−1^/day	Accumulation of the Amyloid Beta (Aβ) in the hippocampus	[54]
28 days	250–300 g	Male Sprague-Dawley rats	HgCl_2_	1148 µg·kg^−1^/day	Fibrosis; asthma; OS	[55]
28 days	42 days old	Male Sprague-Dawley rats	HgCl_2_	1000 µg·kg^−1^	Necrosis; fibrosis in spleen tissue	[56]
30 days	Adult 84 days old	Female Wistar rats	HgCl_2_	First dose 4.6 µg·kg^−1^, subsequent doses 0.07 µg·kg^−1^/day	Presence of ovarian cystic follicles; ovarian OS; glucose/insulin intolerance	[57]
30 days	56 days old	Male Wistar rats	HgCl_2_	First dose 4.6 µg·kg^−1^, subsequent doses 0.07 µg·kg^−1^/day	Anxiety; memory impairment	[58]
30 days	56 days old	Male Wistar strain albino rats	HgCl_2_	1250 µg·kg^−1^	Apoptosis by caspase−3	[59]
45 days (9 h/day)	Adult 56–70 days old 150–200 g	Female Wistar albino rats	Hg vapour	1 mg·m^−3^	Loss of Purkinje cells; deleterious effects on the cerebellum structure	[60]
45 days	90 days old	Male Wistar rats	HgCl_2_	375 µg·kg^−1^/day	Cognitive disorders; OS; cytotoxicity; apoptosis induction	[46]
45 days	150 days old	Male Wistar rats	HgCl_2_	375 µg·kg^−1^/day	Short and long-term memory impairments; motor deficits; accumulation in hippocampus and cortex	[48]
45 days	90 days old	Male Wistar rats	HgCl_2_	375 µg·kg^−1^/day	Motor disorders; cellular death; apoptosis; OS	[47]
7, 14, 28, 42, 56 days	42 days old	Male C57BL/6NJcl mice	MeHg-gluta-thione 1:1	30,000 µg·kg^−1^	Reduction of antioxidant enzyme expression	[61]
56 days	42 days old	Male C57BL/6NJcl mice	MeHg	30,000 µg·kg^−1^	Neuronal degeneration	[62]
60 days	90 days old	Wistar rats	MeHg	40 µg·kg^−1^/day	Alveolar bone loss (in height)	[63]

**Table 3:** Summary of several clinical studies with toxic and neurotoxic effects of Hg exposure in humans.

Exposure Duration	Age of Exposure	Gender	Chemical	Concentration	Major Endpoints	Reference
2 days	40 years old	male	metallic Hg	60 μg·L^−1^ urine	Schizophrenia and inflammatory soft tissue lesions	[104]
7 days	36 years old	male	metallic Hg	300 μg·L^−1^ blood	Abdominal pain, diarrhoea and fever	[105]
14 days	20 years old	female	liquid Hg	510 μg·L^−1^ blood	Fatigue and headache	[106]
14 days	22 years old	female	liquid Hg	129.9 μg·L^−1^ blood	Headache, gingival pain, and numbness in the arms and legs	[106]
14 days	23 years old	female	liquid Hg	430 μg·L^−1^ blood	Fatigue and headache	[106]
14 days	29 years old	male	liquid Hg	544 μg·L^−1^ blood 140 μg·L^−1^ urine	Headache, gingival pain, and numbness in the arms and legs	[106]
14 days	54 years old	female	liquid Hg	518 μg·L^−1^ blood	Fever, cough, night sweats, weight loss, and pain in the extremities	[106]
18 days	36 years old	male	metallic Hg	244 μg·L^−1^ blood	Rash, sore throat, fever, chills, cough and diarrhoea	[107]
26 days	67 years old	male	metallic Hg	1577 μg·L^−1^ blood	Severe pneumonitis, anuria	[108]
50 days (8 h/day)	30 years old	male	metallic Hg	760 μg·L^−1^ urine	Weakness, malaise, excessive diaphoresis, coughing, fever, shortness of breath, diarrhoea, dysphasia, and polyuria	[109]
50 days	53 years old	male	metallic Hg	326 μg·L^−1^ urine	Persistent cough, dyspnoea and insomnia	[109]
60 days (8 h/day)	20 years old	male	metallic Hg	635 μg·L^−1^ urine	Anorexia	[109]
2 years	46 years old	female	metallic Hg	1929 μg·L^−1^ blood	Severe pain, ischemia, erythematous lesions, cyanosis of the left hand	[109]
5 years	33 years old	male	Hg vapour	2.4 μg·L^−1^ urine	Colour vision deficiency	[110]
6 years	37 years old	male	Hg vapour	134.7 μg·L^−1^ urine	Colour vision deficiency	[110]
7 years	42 years old	female	Hg vapour	50 μg·L^−1^ urine	Colour vision deficiency	[110]
8.5 years	36 years old	female	Hg vapour	1.2 μg·L^−1^ urine	Colour vision deficiency	[110]
9 years	34 years old	male	Hg vapour	73.8 μg·L^−1^ urine	Colour vision deficiency	[110]
10 years	43 years old	male	Hg vapour	9 μg·L^−1^ urine	Colour vision deficiency	[110]
12 years	43 years old	male	Hg vapour	56.6 μg·L^−1^ urine	Colour vision deficiency	[110]
12 years	43 years old	male	Hg vapour	66 μg·L^−1^ urine	Colour vision deficiency	[110]
12 years	45 years old	female	Hg vapour	2 μg·L^−1^ urine	Colour vision deficiency	[110]
18 years	36 years old	male	Hg vapour	20 μg·L^−1^ urine	Colour vision deficiency	[110]
not mentioned	21 years old	male	metallic Hg	11,000 μg·L^−1^ serum	Granuloma in the antecubital fossa	[111]
not mentioned	22 years old	male	metallic Hg	3700 μg·L^−1^ blood	Arthro-myalgias, fever, weakness, chest pain (multiple punctuates metallic densities in radiographs)	[112]

**Table 4:** Overview of the chemically synthesized and natural compounds used to minimize neurotoxicity of Hg, dating from 2010 to the present.

Chemically Synthesized or Natural Compound	Hg Type	Experimental Model	Results	Reference
*Celosia argentea* and vitamin E	HgCl_2_	Rats	Protected against the Hg-induced gross, oxidative, cerebral and cerebellar damage	[161]
*Launaea taraxacifolia*	HgCl_2_	Rats	Mitigated the Hg-induced behavioural changes and alteration of the microanatomy of cerebral cortex, hippocampus and cerebellum	[162]
*Citrullus lanatus* seed extract and Vitamin E	HgCl_2_	Rats	Protected against the Hg-induced degeneration of frontal cerebral cortical neurons	[163]
Curcumin	HgCl_2_	Rats	Detoxification and antioxidant effects	[164]
	HgCl_2_	Rats	Ameliorated the behavioural and biochemical alterations in the offspring	[165]
Diallyl sulphide (DAS)	HgCl_2_	Rats	Counteracted the oxidative damage and increased the anti-inflammatory response against the Hg-induced neurotoxicity	[166]
*Dendropanax morbifera Léveille*	Dimethylmercury-(CH_3_)_2_Hg	Rats	Reduced the Hg levels in hippocampal homogenates and increased the activities of antioxidant enzymes	[167]
*Bacopa monniera*	MeHg (CH_3_Hg)	Rats	Protected against the Hg-induced OS	[168]
Grape Seed Proanthocyanidin Extracts	CH_3_Hg	Rats	Counteracted the oxidative damage	[169]
Vitamin K	CH_3_Hg	Primary cultured neurons from the cerebella of rat pups	Protected the neurons against Hg cytotoxicity	[170]
Sodium selenite (Na_2_SeO_3_)	CH_3_Hg	Rats	Modulated the autophagic and apoptotic milieu of the cells via inhibiting the ROS-mediated apoptosis	[171]
NAC	CH_3_Hg	Rats	Reduced the Hg-induced toxicity in the developing rat hippocampus	[172]

## Data Availability

The datasets used and analysed during the current study are available from the corresponding author upon reasonable request.
